# Maternal and early childhood factors associated with asthma and obesity in children aged 6 to 7 years: a case control study

**DOI:** 10.31744/einstein_journal/2022AO5609

**Published:** 2022-02-08

**Authors:** Bruna Becker da Silva, Jane da Silva, Jefferson Luiz Traebert, Aline Daiane Schlindwein

**Affiliations:** 1 Universidade do Sul de Santa Catarina Palhoça SC Brazil Universidade do Sul de Santa Catarina, Palhoça, SC, Brazil.; 2 Universidade Federal de Santa Catarina Florianópolis SC Brazil Universidade Federal de Santa Catarina, Florianópolis, SC, Brazil.; 3 Secretaria de Estado da Saúde de Santa Catarina Florianópolis SC Brazil Secretaria de Estado da Saúde de Santa Catarina, Florianópolis, SC, Brazil.

**Keywords:** Asthma, Overweight, Obesity, Child

## Abstract

**Objective:**

To determine the maternal and early childhood factors associated with asthma and obesity in children aged 6 to 7 years.

**Methods:**

A case-control study conducted with children aged 6 to 7 years. Applications with questions about asthma symptoms in the last 12 months, maternal and childhood data in the first 2 years of life, and anthropometric data were collected. Children who presented asthma symptoms were considered as cases and those without asthma symptoms were considered as controls, later divided into two subgroups that were eutrophic or overweight/obesity. Logistic regression was performed to estimate the association between asthma symptoms (adequate weight and overweight/obesity) and gestational and personal factors, calculating odds ratio and 95% confidence interval (95%CI). Values of p<0.05 were considered significant.

**Results:**

Two hundred and one children were evaluated, 25.4% had asthma symptoms, 37.2% of them were overweight/obesity. Waist circumference, triceps skinfold, and body mass index were higher in the group with overweight/obesity asthma symptoms compared to no asthma symptoms (p<0.05). Factors significantly associated with asthma and overweight/obesity symptoms included: the maternal history of asthma (odds ratio of 3.73; 95%CI: 1.10-12.6) and hypertension during pregnancy (odds ratio of 3.29; 95%CI: 1.08-9.94).

**Conclusion:**

Maternal history of asthma and hypertension during pregnancy increased the chances of children, at 6 and 7 years of age, having symptoms of asthma and obesity.

## INTRODUCTION

Asthma is a chronic inflammatory disease of the airways, and its main symptoms are wheezing, dyspnea, and cough, which vary over time and in intensity, with variable limitation of expiratory airflow.^(1)^ Many studies have indicated an increase in the prevalence of asthma in childhood during the last decades, mainly in developing countries. Brazil is positioned in sixth place in the rank of prevalence of asthma, and the prevalence of asthma symptoms is diagnosed in over 20% of children, and youth between 7 and 14 years of age.^(2)^

According to the World Health Organization (WHO), overweight and obesity are characterized by the accumulation of body fat that can damage health, and the latter is considered a chronic disease.^(3)^ They are associated with several health risks, because of their correlation with various metabolic complications, cardiovascular diseases, and asthma.^(3)^ In Brazil, it is estimated that 33.5% of the child population is overweight.^(4)^

The relationship between asthma and overweight/obesity has been explored in the published literature, but without reaching a consensus, and it is still a subject of new investigations. It is known that overweight can precede asthma symptoms.^(5)^ In a longitudinal study that followed more than 4,000 children without asthma for 14 years, it was found that body mass index (BMI) ≥85^th^ percentile at age 2 to 3 years increased the risk for developing asthma, especially in boys.^(6)^

The first 1,000 days of life include from the time of conception to 2 years of age (270 days of gestation and 365 days of the first year of life plus 365 days of the second year of life).^(7)^ This period of life is crucial for child growth and development, because it is when some healthy habits and choices are established, given their impact on health and disease indicators throughout the life of this individual.^(7)^Some studies have shown that this period plays an important role in the prevention and also in the appearance of certain diseases in the future, including asthma, and overweight/obesity.^(8,9)^

Although the association of both diseases is still under discussion, it is known that overweight/obesity worsens asthma symptoms.^(5)^However, the identification of factors that contribute to the development of asthma symptoms and overweight/obesity in children at 6 and 7 years of age should be investigated during the first 1,000 days of life as an attempt to better understand the association between these diseases.

## OBJECTIVE

To determine the maternal and early childhood factors associated with asthma and obesity in 6- to 7-year-old children.

## METHODS

Population-based case-control study nested in a retrospective cohort^(10)^ with children aged 6 to 7 years in the city of Palhoça (SC), conducted from August to December 2016.

A sample calculation was performed using the Open Source Epidemiologic Statistics for Public Health (OpenEpi) 3.03a program from the Rollins School of Public Health at Emory University, Atlanta, USA. The minimum sample size with 80% power, 95% confidence level, 1:3 ratio (case: control), considering 50% as the percentage of exposed controls and 20% the cases with exposure, was 31 cases and 92 controls. Taking into account the possibility of losses and refusals of 20%, the total minimum sample was 148 students.

Palhoça had 57 elementary schools. From these, 27 public and 12 private schools were randomly selected. Subsequently, a proportional stratified probability sampling was carried out to determine the number of students in each school. As inclusion criteria, children had to be born in 2009, residents, and enrolled in schools in the city. The study criteria included 1,900 children, whose data were obtained from the municipality’s education department. After obtaining these data, the consent form was sent via school to the parents/guardians, and those who agreed to participate in the study returned the complete consent form to the school. Children who refused to perform any of the anthropometric data measurements, or who were wheelchair users or had a cast on a limb, were excluded from the study. Children whose mothers or caretakers were not found at home during three consecutive visits (one of the visits was made on the weekend) to fill out the questionnaire, as well as children who were not at school at the time of the anthropometric evaluation were considered lost.

Asthma symptoms were characterized using the International Study of Asthma and Allergies in Childhood (ISAAC) questionnaire, which has been translated and cross-culturally adapted for Brazilian Portuguese.^(11)^ The ISAAC has been used to identify asthma symptoms in children during the last 12 months. According to the initial validation of the ISAAC by Solé et al.,^(11)^ the question regarding wheezing in the past 12 months was the one that presented the highest sensitivity and specificity for its use in epidemiological studies worldwide. The criterion for inclusion of a child in the case group was a positive response from the mother/guardian to the question in the ISAAC questionnaire:^(11)^In the past 12 months, has your child had wheezing? The anthropometric evaluation and the same question with a negative answer were used to select the controls.

Community Health Workers (CHWs) were invited to apply the questionnaires; they had undergone a training course in epidemiological research with emphasis on data collection, in order to avoid possible measurement bias during the application of the questionnaires.^(10)^ Parents who authorized the child’s participation in the study received a home visit from the CHW to fill out the questions on the questionnaire, which was previously structured and tested in a pilot study.^(10)^ The questions obtained were about socioeconomic conditions, diet in the first 2 years of life, gestational data (how many kilos gained, diabetes, hypertension, and smoking), family history of asthma, and perinatal data that were obtained from the health card or by verbal report from the mother/guardian.

For the anthropometric measurements, the procedures were: body weight was measured using a calibrated digital scale (model BAL 150-3037, Powner), with precision of 100g; height was measured with a portable stadiometer (model EST_PORT_PC, Avanutri), ranging from 20cm to 210cm, with precision of 1mm. The triceps skinfold thickness (TSF) was also measured with an adipometer (Traditional Clinical model, Cescorf), with a scale of 1mm and reading range of 75mm. Arm circumference (AC), waist circumference (WC), and neck circumference (NC) were measured using a tape measure (model 201, Seca), with a measurement range of up to 205cm and a scale of 1mm. Anthropometric data on weight, height, TSF, AC, NC, and WC were measured by a nutritionist according to standardized procedures in an appropriate room.^(12-14)^

Body mass index was used to classify the children as normal weight and overweight/obesity according to age- and sex-specific cut-off points, considering the growth curves adopted by the Ministry of Health.^(12)^ Overweight/obesity children were considered those whose BMI > 85^th^ percentile, according to the Centers for Disease Control standardized growth charts for age and sex used in other studies.^(6,15)^The first 201 children who had the questionnaires duly filled out and the anthropometric data collected were randomly selected. Of these, 25.4% were considered cases and 75.6% were controls. The children were divided into two groups: with asthma symptoms and without asthma symptoms; subsequently, they were divided into four subgroups: eutrophic asthmatics, obese asthmatics, eutrophic non-asthmatics, and obese non-asthmatics.

The data obtained were compiled and exported to Microsoft Excel, and these were submitted to statistically analyzed using the IBM SPSS software, version 18.0. Continuous variables were presented as mean and standard deviation, while categorical variables were presented as simple and relative frequencies. The Kolmogorov-Smirnov normality test was performed to evaluate the normality hypothesis of the distribution of continuous variables. Student’s *t*-test or Mann-Whitney test assessed the differences in the variables between those with appropriate weight and those with overweight/obesity. The χ^2^ test was used to assess the differences between categorical variables. Analysis of variance (ANOVA) or the Kruskal-Wallis test was used to check the differences in variables between the groups with asthma symptoms and those without asthma symptoms.

Subsequently, the relationships of asthma symptoms with measures of overweight/obesity were tested with multivariate logistic regression analysis in order to observe the independence of the associations, adjusting for potential confounding factors. Data are presented as odds ratios (OR) and 95% confidence intervals (95%CI). All statistical tests were two-dimensional, and data were considered statistically significant, with p value of <0.05. Body mass index data were analyzed with the WHO application Anthro, and the other anthropometric data with the aid of Avanutri Online software. This study was approved by the Research Ethics Committee of the *Universidade do Sul de Santa Catarina* (UNISUL), # 897.811 and CAAE: 38240114.0.0000.5369.

## RESULTS

We evaluated 201 children, 25.4% of whom presented asthma symptoms and 30.8% of whom were overweight/obesity. Among the children with asthma symptoms, 37.2% were overweight/obesity, and, among those without asthma symptoms, 28.6% were overweight/obesity. Table 1 shows the sociodemographic and anthropometric characteristics. In both groups, most of the children were white and studied in public schools. There was no significant change when comparing eutrophic and obese children with asthma symptoms regarding: gender, ethnicity, type of school, parents’ education, and family income. However, 13.1% of browns were recorded among the eutrophic children without asthma symptoms compared with 2.3% among those with overweight/obesity, and 0.9% of them were indigenous (p=0.008). In addition, obese non-asthmatic children had higher family income (R$ 2,32±1.447,5 v*ersus* R$ 2.151,40±1.097,76, p=0.033) when compared with eutrophic non-asthmatic children. When these factors were analyzed among the four groups, there was no significant change (p>0.05).

**Table 1:** Sociodemographic and anthropometric characteristics, according to the presence of asthma symptoms and overweight/obesity in children aged 6 to 7 years in the municipality of Palhoça - Santa Catarina, 2016

Evaluated data	Eutrophic asthmatic (n=32)	Obese asthmatic (n=19)	p value*	Eutropic non-asthmatic (n=107)	Obese non-asthmatic (n=43)	p value*	p value^†^
Sex			0.524				0.600
Men	16 (50)	11 (57.9)		49 (45.8)	24 (55.8)	0.270	
Women	16 (50)	8 (42.1)		58 (54.2)	19 (44.2)		
Ethnicity			0.528			0.008	0.063
White	24 (75)	12 (63.1)		87 (81.3)	40 (93)		
Black	1 (3.1)	3 (15.8)		5 (4.7)	2 (4.7)		
Brown	7 (21.9)	3 (15.8)		14 (13.1)	1 (2.3)		
Asian		1 (5.3)					
Indigenous				1 (0.9)			
Type of school			0.894			0.481	0.284
Public	29 (90.6)	17 (89.5)		92 (86)	35 (81.4)		
Private	3 (9.4)	2 (10.5)		15 (14)	8 (18.6)		
Parents’ education, years							
Mother	8.23±2.77	9.11±2.47	0.323	9.19±3.42	8.19±3.17	0.338	0.265
Father	8±3.23	7.36±2.59	0.478	8.11±3.73	8.23±3.34	0.539	0.270
Family income, R$	2.272,8±1.322,6	2.589,39±1.068,34	0.495	2.151,40±1.097,76	2.472,32±1.447,65	0.033	0.267
Weight, kg	22.87±2.79	35.33±8.75	0.001	23.10±2.91	31.30±3.94	0.001	0.034
Height, m	1.22±0.05	1.28±0.05	0.001	1.23±0.05	1.27±0.05	0.001	0.908
Body mass index, kg/m^2^	15.39±1.16	21.36±4.20	0.001	15.16±1.25	19.23±1.71	0.001	0.027
Waist circumference, cm	54.92±3.61	69.55±12.01	0.001	54.87±3.55	65.12±5.89	0.001	0.049
Neck circumference, cm	25.77±1.17	28.39±2.13	0.001	25.44±1.71	28.31±2.19	0.001	0.214
Arm circumference, cm	16.50 (14-19.50)	20.5 (18-29)	0.001	17 (14-21)	20 (15-29)	0.001	0.133
Triceps skinfold, mm	15 (10-20)	20 (13-35)	0.001	15 (5-25)	15 (10-25)	0.001	0.046

* p values obtained using Mann-Whitney test or Student’s *t*-test; ^†^ p values obtained using analysis of variance or Kruskal-Wallis test, for comparison between the groups with asthma and those without asthma.

Results expressed as n (%), mean ± standard deviation or mean (minimum-maximum).

Regarding the anthropometric and body composition data, among the children with asthma symptoms, 37.2% were overweight/obesity, and among those without asthma symptoms, 28.7% were overweight/obesity; p=0.034), BMI (21.36±4.20kg/m^2^; p=0.027), WC (69.55±12.01cm; p=0.049), and TSF (20 [13-35]mm; p=0.046), compared with non-obese asthmatic children. However, when the nutritional profile was evaluated, overweight/obesity children had higher values for weight, height, BMI, AC, WC, NC, and TSF than eutrophic children, both in children with asthma symptoms and those without symptoms (p<0.001).

Perinatal factors were also evaluated (Table 2). In children with asthma symptoms, mothers reported during pregnancy having hypertension independent of the nutritional status of the child (21.9% eutrophic and 26.3% overweight/obesity; p=0.027), when compared with children without asthma symptoms (7.5% and 18.6%). However, when compared with children with asthma symptoms, mothers of obese asthmatic children gained more weight during pregnancy (19.44±10.61kg *versus* 7.75±2.63kg; p=0.008) compared to mothers of eutrophic asthmatic children. However, obese non-asthmatic children had higher gestational age (39.34±2.74 *versus* 38.98±2.17, p=0.036) and more family history of asthma (60.5% *versus* 32.7%; p=0.002) compared with eutrophic non-asthmatic children.

**Table 2:** Perinatal factors according to the presence of asthma symptoms and overweight/obesity in children aged 6 to 7 years in the municipality of Palhoça - Santa Catarina, 2016

Evaluated data	Eutrophic asthmatic (n=32)	Obese asthmatic (n=19)	p value*	Eutropic non-asthmatic (n=107)	Obese non-asthmatic (n=43)	p value*	p value^†^
Maternal age, years	29±7.83	30.37±17.76	0.825	25.33±4.09	28.17±10.04	0.114	0.147
Maternal ethnicity			0.356			0.541	0.496
White	23 (71.9)	10 (52.6)		81 (75.8)	35 (81.4)		
Black	2 (6.2)	5 (26.3)		11 (10.3)	2 (4.7)		
Brown	7 (21.9)	3 (15.8)		12 (11.2)	5 (11.6)		
Asian		1 (5.3)		2 (1.8)	1 (2.3)		
Indigenous				1 (0.9)			
Gestational weight gain, kg	7.75±2.63	19.44±10.61	0.008	11.16±5.67	15.58±11.38	0.249	0.286
Gestational age, weeks	38.50±2.46	38.74±1.93	0.964	38.98±2.17	39.34±2.74	0.036	0.115
Gestational diabetes			0.171			0.678	0.188
Yes	1 (3.1)	3 (15.8)		4 (3.7)	1 (2.3)		
No	31 (96.9)	16 (84.2)		103 (96.3)	42 (97.7)		
Gestational hypertension			0.647			0.092	0.027
Yes	7 (21.9)	5 (26.3)		8 (7.5)	8 (18.6)		
2No	25 (78.1)	14 (73.7)		99 (92.5)	35 (81.4)		
Maternal smoking during pregnancy			0.076			0.697	0.854
Yes	6 (18.8)	2 (10.5)		17 (15.9)	8 (18.6)		
No	26 (81.2)	17 (89.5)		90 (84.1)	35 (81.4)		
Maternal history of asthma			0.526			0.447	0.119
Yes	6 (18.8)	1 (5.3)		6 (5.6)	4 (9.3)		
No	26 (81.2)	18 (94.7)		101 (94.4)	39 (90.7)		
Family history of asthma			0.143			0.002	0.153
Yes	23 (71.9)	9 (47.4)		35 (32.7)	26 (60.5)		
No	9 (28.1)	9 (47.4)		70 (65.4)	17 (39.5)		
Do not know		1 (5.2)		2 (1.9)			

* p values obtained using Mann-Whitney test or Student’s *t-*test; ^†^ p values obtained using analysis of variance or Kruskal-Wallis test, for comparison between the groups with asthma and those without asthma.

Results expressed as mean ± standard deviation or n (%).

When the four groups were evaluated regarding the food history in the first two years of life, the obese non-asthmatic children consumed more processed food (27.9%; p=0.015) compared with the other groups. Most children were breastfed, regardless of whether they had asthma symptoms or not. However, when the children with asthma symptoms were evaluated, the obese asthmatic children had the shortest period of exclusive breastfeeding (3.31±2.35 *versus* 5.33±1.2; p=0.034), as well as using more infant formula before the sixth month (73.7% *versus* 43.8%; p=0.049), unlike the eutrophic asthmatic children. In children without asthma symptoms, there was no significant difference when comparing the eutrophic and obese non-asthmatic groups (p>0.05) (Table 3).

**Table 3:** Dietary history, according to the presence of asthma symptoms and overweight/obesity in children aged 6 to 7 years in the municipality of Palhoça - Santa Catarina, 2016

Evaluated data	Eutrophic asthmatic (n=32)	Obese asthmatic (n=19)	p value*	Eutropic non-asthmatic (n=107)	Obese non-asthmatic (n=43)	p value*	p value^†^
Breastfeeding	28 (87.5)	14 (73.7)	0.257	99 (92.5)	37 (86)	0.392	0.080
Period of exclusive breastfeeding, months	5.33±1.2	3.31±2.35	0.034	4.74±2.45	4.26±2.08	0.313	0.523
How long was breastfeeding, months	18.26±13.89	22±21.19	0.748	16.14±13.25	16.77±17.60	0.850	0.369
Age of introduction of milk and dairy products, months	7.30±3.96	8.31±3.35	0.455	8.26±6.09	7.91±3.26	0.626	0.116
Age of fruit introduction, months	6 (3-20)	6 (4-6)	0.114	6 (1-24)	6 (2-25)	0.200	0.062
Fruit			0.601			0.399	0.015
Unprocessed	29 (90.6)	18 (94.7)		84 (78.5)	30 (69.8)		
Industrialized baby food	3 (9.4)	1 (5.3)		21 (19.6)	12 (27.9)		
Do not remember				2 (1.9)	1 (2.3)		
Use of dairy foods before the sixth month^‡^	14 (43.8)	14 (73.7)	0.049	55 (51.4)	28 (65.1)	0.127	0.935
Use of milk derivatives	29 (90.6)	18 (94.7)	0.597	97 (90.7)	41 (95.3)	0.231	0.671
Use of sugar	15 (46.9)	5 (26.3)	0.141	40 (37.4)	11 (25.6)	0.152	0.564
Use of potentially allergenic foods before 1 year^§^	28 (87.5)	17 (89.5)	0.832	98 (91.6)	39 (90.7)	0.798	0.427

* p values obtained using Mann-Whitney test or Student’s *t*-test; ^†^ p values obtained using analysis of variance or Kruskal-Wallis test, for comparison between asthma and non-asthmatic groups; ^‡^ dairy foods: cow’s milk or infant formula; ^§^ potentially allergenic foods: egg, peanut, cow’s milk, and seafood.

Results expressed as n (%), mean ± standard deviation or mean (minimum-maximum).

Table 4 presents the results of the raw and adjusted multivariate logistic regression analyses evaluating the associations between obese asthmatics. In addition, children whose mothers had hypertension during pregnancy were 3.29 times more likely to have asthma symptoms and overweight/obesity than those who were eutrophic controls (OR=3.29; 95%CI: 1.08-9.94; p=0.035). Concerning the other factors, no statistically significant difference was observed.

**Table 4:** Multivariate analysis by logistic regression for asthma symptoms and overweight/obesity in relation to children without asthma symptoms and eutrophic

Factors	n (%)	Raw analysis		Adjusted analysis	
	
		Odds ratio (95%CI)	p value	Odds ratio (95%CI)	p value
Sex					0.791
Men	11/60 (57.9)	1.18 (0.53-2.60)	0.676	1.12 (0.45-2.77)	
Women	8/66 (42.1)	1		1	
Pregnancy hypertension			0.034		0.035
Yes	5/13 (26.3)	3.33 (1.09-10.1)		3.29 (1.08-9.94)	
No	14/113 (73.7)	1		1	
Natural fruit			0.221		0.452
<Yes	18/30 (42.1)	1.73 (0.71-4.19)		1.56 (0.48-5.00)	
≥No	10/94 (52.6)	1		1	
Maternal history of asthma					
Positive	1/7 (28.5)	4.25 (1.01-17.8)	0.048	3.73 (1.10-12.6)	0.034
Negative	14/115 (71.5)	1		1	

Model adjusted for age and body mass index.

OR: odds ratio; 95%CI: 95% confidence interval.

## DISCUSSION

The results of this study show that the presence of asthma symptoms and overweight/obesity in children aged 6 to 7 years is significantly associated with some determinants in the first 1,000 days of life, such as maternal history of asthma and hypertension during pregnancy. The prevalence of obese asthmatic children was 37.2% in the present study. Roncada et al.,^(16)^evaluated the anthropometric status and its relation to asthma in 8- to 16-year-old schoolchildren in public schools in the city of Porto Alegre (RS), and observed a prevalence of 34.8% of schoolchildren with asthma and overweight/obesity, which was slightly lower than our findings. However, this difference can be justified by the sample size, which, if in the present study were larger, the data found could be similar.

The prevalence of obesity in children with asthma has been lower in other studies. Silva et al.,^(15)^*e.g.,* found that 8.3% of the students with wheezing were obese; in the case of Zacaron et al.,^(17)^ In contrast, Tai et al.,^(18)^ found lower prevalence of overweight (13.7%) and obesity (5.7%) in children with asthma.

As expected, the nutritional profile of the eutrophic children was significantly different from that of the overweight/obesity children in both groups, with/without asthma symptoms. However, when comparing children without asthma symptoms to those with asthma symptoms, obese asthmatics had significantly higher weight, BMI, WC, and TSF values. These results have also been demonstrated in other studies,^(18,19)^ with possible explanations being genetic, lifestyle, and environmental factors,^(18)^ as well as decreased physical activity.^(20)^ Children with asthma tend to be more sedentary, since physical exercise can lead to bronchospasm, which manifests as cough, wheezing, and dyspnea, in different degrees of intensity.^(21)^

The demographic characteristics of this study did not differ significantly regarding the presence of asthma symptoms and/or overweight/obesity. On the other hand, various studies that have specifically evaluated the association of socioeconomic and racial-ethnic indicators with asthma have indicated a higher prevalence of asthma in males in the first years of life,^(22)^ lower level of education of the parents of children with asthma,^(22,23)^ lower family income,^(23)^ and black ethnicity.^(24)^ Perhaps due to the small number of participants, it was not possible to show these associations in the present study.

Regarding gestational data, mothers of obese asthmatic children had greater weight gain compared to mothers of eutrophic asthmatic children (p<0.01). In the literature, there are data showing that high weight gain during pregnancy contributes to the increased risk of asthma or wheezing in children, due to the specific inflammatory state related to adiposity, which is associated with a chronic inflammatory state, in which there are high levels of inflammatory cytokines, such as leptin.^(25)^ Leptin is found at high levels in the circulation of obese pregnant women, which incurs in its higher levels in the cord blood and can lead to a greater chance of asthma development, as well as childhood overweight/obesity.^(26)^ This could be observed in the present study, since the mothers with greater weight gain during pregnancy had overweight/obesity children.

Mothers of children with asthma symptoms significantly more often had gestational hypertension than mothers of children without asthma symptoms (p<0.05). Studies evidencing this relationship are scarce.^(27,28)^ Stick et al.,^(27)^ have demonstrated that newborns born to mothers with hypertension before or during gestation have a reduction in pulmonary function similar to those exposed to maternal gestational smoking. This is a known risk factor for decreased lung function, early wheezing, and development of asthma in children at 6 years of age.^(29,30)^ However, prospective longitudinal studies are necessary in order to make inferences regarding this relationship.

Family history of asthma was not frequently observed in children with asthma symptoms, regardless of nutritional profile. Surprisingly, obese non-asthmatic children had significantly more positive family history of asthma compared to eutrophic children (p<0.01). This result might have relevance if it is considered that overweight/obesity and family history of asthma are risk factors for asthma.^(8)^

In this case, it is speculated that, even in the group of children without asthma symptoms, it is possible that the disease will occur, since asthma is associated with genetic factors, and the individual who is born with this propensity, when exposed to certain stimuli, will have an inflammatory reaction and bronchial closure, which can occur in any phase of life. However, only longitudinal follow-up would be able to answer this question.

Exposure to cigarette smoking in utero and during the postnatal period has been associated with wheezing, asthma in early childhood, and respiratory morbidity. However, maternal smoking, in this study, did not differ significantly with respect to the presence of asthma symptoms or not. One possible explanation is that most mothers of children with asthma symptoms did not smoke during pregnancy, unlike other findings.^(31)^

One of the factors of interest as a possible chance of developing asthma in childhood is related to nutrition, especially in the first 1,000 days of life. Obese asthmatic children had shorter duration of exclusive breastfeeding compared with eutrophic asthmatics, as well as greater use of infant formula before 6 months (p<0.05). To break this cycle, many mothers introduce cow’s milk and its derivatives early. These foods have higher amounts of carbohydrates, proteins, and fats than breast milk, and the use of bottle feeding induces supplementation with sugar and flour, which can contribute to weight gain.^(32)^

However, some limitations apply to this study, since it is a case-control observational study and has a reduced number of participants, which may not show some differences that, by chance, would exist. The use of a questionnaire with questions that depend on the memory of the mother and/or legal responsible may generate information bias, but studies have shown that parents are able to remember diseases or occurrences with their children. However, epidemiological studies using questionnaires are low-cost and extremely useful for generating information,^(33)^leading, consequently, to the development of improvements in the Brazilian Public Health System (SUS - *Sistema Único de Saúde*).

## CONCLUSION

The prevalence of asthma and obesity in children at 6 and 7 years of age was high. Perinatal and dietary factors in the first 2 years associated with asthma and obesity were hypertension and gestational weight gain, higher gestational age, family history of asthma, shorter breastfeeding period, and higher use of infant formula. Therefore, it is important to introduce clinical guidelines as well as public health recommendations for the population.
